# eDEM-CONNECT: agitation ontology for the intelligent support of informal caregivers of people with dementia

**DOI:** 10.3389/fragi.2026.1780260

**Published:** 2026-05-01

**Authors:** Sumaiya Suravee, Christiane Pinkert, Iris Hochgraeber, Margareta Halek, Bernhard Holle, Kristina Yordanova

**Affiliations:** 1 Institute of Data Science, University of Greifswald, Greifswald, Germany; 2 German Centre for Neurodegenerative diseases (DZNE), Witten, Germany; 3 CIS - Cellitinneninstitut für Qualitätssicherung, Seniorenhaus GmbH der Cellitinnen zur hl. Maria, Cologne, Germany; 4 School of Nursing Science, Faculty of Health, Witten Herdecke University, Witten, Germany

**Keywords:** agitation, dementia, digital caregiver support, intelligent solutions, non-clinical interventions, ontology

## Abstract

People with dementia (PwD) face cognitive decline, placing added stress on family caregivers. Challenging behaviour, such as agitation, is one of the prominent behaviours exhibited by PwD, and family caregivers are often faced with the challenge of finding an appropriate intervention strategy to cope with it. To address this problem, current research focuses on developing digital solutions for the support of unprofessional caregivers, allowing them to ease the stress factor while dealing with agitation. A major challenge in any digital solution is the required domain knowledge. This knowledge includes information about the types of agitated behaviour, living and socio-economic conditions of the PwD and non-pharmaceutical interventions, which the caregiver can apply. We refer to this structured knowledge as an ontology. This study focuses on the development of the eDEM-Connect Ontology: Ontology of Dementia-related Agitation and Relationship between Informal Caregivers and Persons with Dementia (EDEM-CONNECTONTO) as the formalised domain knowledge for providing adequate support to caregivers. The knowledge is elicited through a systematic literature review, analysis of existing ontologies, workshops with experts, and interviews with informal caregivers. EDEM-CONNECTONTO consists of 252 Concepts, 16 relations, and 241 individuals. The ontology is implemented in the Web Ontology Language (OWL) and validated with the Protégé ontology development software. The results from the evaluation show that it meets the standard for biomedical ontologies. Furthermore, EDEM-CONNECTONTO is applied in several tasks related to the development of digital support systems for caregivers of PwD, demonstrating its practical applicability within the domain. The proposed ontology provides a structured semantic foundation for ontology-guided data annotation, knowledge graph construction, and GraphRAG-based caregiver-support applications. By formally modelling types of agitation, causes, consequences, PwD-caregiver relations, and non-pharmacological interventions, EDEM-CONNECTONTO enables explainable digital tools that support informal caregivers in identifying agitation patterns and selecting appropriate care strategies, thereby contributing to improved caregiver support and reduced caregiving stress.

## 1 Introduction

Dementia affects over 55 million people worldwide (World Health Organization, 2023). It leads to limitations in cognitive abilities, such as declining memory as well as changes in behaviour or emotional control (World Health Organization, 2023). The most common form of behavioural change in people with dementia (PwD) is agitation (Thyrian et al., 2015). Agitation is a persistent or frequently recurring behaviour that is primarily manifested in excessive motor activity, leading to significant impairments in interpersonal relationships and the ability to accomplish everyday tasks (Cummings et al., 2015). In Germany, about 70% of very old PwD live at home and are mainly cared for by relatives and outpatient care services (Brijoux and Zank, 2023). Dealing with agitated behaviour is a major challenge for formal and informal carers. Agitation can lead to early placement of the PwD in a nursing home Yaffe et al. (2002), cause stress to the family caregivers George et al. (2020), and threaten the stability of home-based care arrangements. Behavioural problems also hurt the quality of the dyadic relationship (De Vugt et al., 2003). Disturbed communication is a major challenge for family caregivers and leads to a negative perception of the relationship (Pozzebon et al., 2016). Therefore, the dyadic relationship may influence the development of agitation, which can impact the quality of life of the individuals concerned (Luiu et al., 2020). To support families in managing agitated behaviour, needs-based situation-specific information and support services are required. Although both formal and informal services are available, their utilisation is low, and the reasons are diverse (Stephan et al., 2018; Vecchio et al., 2016; Michalowsky et al., 2019). Family caregivers often cannot recognise which information is important for them or have difficulties applying it to their situation. Family caregivers primarily contact help services via an online search engine, which often finds irrelevant, confusing or outdated content. Thus, there is a strong urge for user-centred digital support systems that could potentially reduce the burden of the caregiver. The first step in developing such systems is the collection and formalisation of the relevant domain knowledge. To address this problem, we developed eDEM-Connect: Ontology of Dementia-related Agitation and Relationship between Informal Caregivers and Persons with Dementia (EDEM-CONNECTONTO), which allows incorporating reliable and validated knowledge in modern machine learning systems, from domain-specific entity recognition and relation extraction to chatbot systems such as ChatGPT (De Angelis et al., 2023). Thus, it potentially improves the performance of situation-aware user-centred systems in high-risk applications. The EDEM-CONNECTONTO^1^ is publicly available in Bioportal Geller et al. (2018), the largest repository for publishing biomedical ontologies, hosting more than 715 ontologies and continuously growing. The EDEM-CONNECTONTO has been used for different applications, for example, as a codebook for data annotation Suravee et al. (2022), which is later used for domain-specific named entity recognition and relation extraction tasks Suravee et al. (2025a), Suravee et al. (2025b), as a knowledge base for classical rule-based and probabilistic chatbots Boiting et al. (2023), and as a knowledge base for modern chatbots that rely on large language models (LLMs) and retrieval augmented generation (RAG) Lewis et al. (2020) to provide more reliable information to the user.

An ontology provides a formal representation of knowledge within a shared domain by defining its core concepts and the relationships among them. It specifies classes, instances, attributes and logical constraints, such as rules and axioms, thereby enabling a structured, reusable and interoperable model of knowledge (Gruber, 1993). Ontologies serve as conceptual domain models that capture a field of knowledge through a set of concepts and their interrelations, reflecting real-world entities and their associations (Globa et al., 2018). When applied to real-world data, an ontology enables the construction of a knowledge graph in which concepts and relationships are represented as interconnected nodes and edges.

Within the Semantic Web framework, ontologies form a core component of the World Wide Web Consortium (W3C) standards stack (W3C, 2013). They build on semantic technologies such as the Resource Description Framework (RDF) and description logics to represent knowledge in a machine-interpretable language, namely as the Web Ontology Language (OWL) (W3C, 2013; Globa et al., 2018). By embedding semantics within data, ontologies enable machines not only to store and retrieve information but also to interpret, integrate, and reason over it (Lan et al., 2022). Ontologies may be developed using top-down, bottom-up or middle-out methodologies, as well as through the reuse of existing ontological resources (Musen, 2015). In a top-down approach, modelling begins with general concepts that are progressively refined into more specific classes, whereas the bottom-up approach starts with highly specific concepts that are subsequently abstracted into broader categories. These complementary strategies support flexible and systematic ontology development across diverse application domains (Musen, 2015).

In recent years, ontologies have been widely adopted in the health domain to support the structured representation, integration, and analysis of complex biomedical data (Hollis et al., 2019; Cimino, 1998). For instance, the Gene Ontology defines concepts representing gene functions and the relationships among them (Consortium, 2019). The Systematised Nomenclature of Medicine–Clinical Terms (SNOMED CT) ontology provides a comprehensive clinical healthcare terminology that contains over 352,567 concepts (El-Sappagh et al., 2018). The ontology of Logical Observation Identifier Names and Codes (LOINC) McDonald et al. (2003) comprising 281,878 classes and 140 properties, provides a standardised set of names and codes for identifying laboratory tests and clinical measurements. Research has also been conducted in the dementia domain. For instance, Roantree et al. (2016) published the In-MINDD ontology, which models the risk factors that can cause dementia. In Meditskos (2015), the Dem@Care Lab ontology is presented as a formal representation of the experimental protocol for dementia assessment. It supports early diagnosis, facilitates the monitoring of disease progression, and contributes to maintaining independent living among PwD. However, there are a few non-pharmacological ontologies available in this domain. In Fook et al. (2006), the authors presented an ontology-based context model that can handle agitation behaviour in PwD in a context-aware manner. Zhang et al. (2020) developed a comprehensive ontology focused on non-pharmacological interventions aimed at managing agitated behaviours in PwD. The Alzheimer’s Disease Ontology (ADO) Malhotra et al. (2014) provides an extensive representation of knowledge related to Alzheimer’s disease, including its underlying causes, clinical manifestations, therapeutic interventions, and associated medical implications. The User Profile ontology Skillen et al. (2012) captures the person-related factors influencing one’s behaviour and their changes with the progression of dementia. It addresses the issues on outdoor mobility of PwD. The DREAMDNPTO Zhang et al. (2024) consists of 1258 unique classes which are focused only on dementia-related emotional and mood disturbance non-pharmacological interventions. The Situation-Aware Navigation Assistance for Dementia Patients using Causal Behaviour Models (SinDem) ontology Yordanova et al. (2017) contains knowledge relevant to supporting the outdoor mobility of people in the early stages of dementia. It has been used to annotate the outdoor behaviour of PwD during a guided walk recorded with a camera and a variety of sensors. However, to the best of our knowledge, no existing non-pharmacological ontology provides a comprehensive representation that captures in detail the agitation behaviours of PwD, the underlying causes that trigger such behaviours, their consequences for both individuals and their social environment, and the dyadic relationship between PwD and their informal caregivers. As a result, digital caregiver-support systems often struggle to incorporate contextual knowledge that reflects real-world caregiving situations. To address this gap, we introduce EDEM-CONNECTONTO, a structured and peer-reviewed ontology that formally models agitation-related behaviours in PwD, the factors contributing to such behaviours, their social and interpersonal consequences, and relevant non-pharmacological interventions. By providing a structured semantic representation of these elements, EDEM-CONNECTONTO establishes a foundation for ontology-driven digital support systems that can assist informal caregivers and nursing professionals in understanding agitation triggers and selecting appropriate intervention strategies. Beyond conceptual modelling, EDEM-CONNECTONTO serves as a practical semantic backbone for multiple computational applications. It is operationalised within (i) a GraphRAG-based dementia caregiver-support chatbot, (ii) the development of a domain-specific dementia dataset guided by ontology-based annotation schemes, (iii) experimental pipelines for named entity recognition and relation extraction tasks, and (iv) the construction of eDEM-KG, an ontology-driven knowledge graph representing dementia-related agitation events. In summary, this study contributes to research in dementia care informatics and ontology-based digital support systems through the following key contributions:

- This work introduces EDEM-CONNECTONTO, a formally structured ontology that captures agitation-related behavioural phenomena in PwD, including behavioural manifestations, contextual triggers, and caregiving interactions.
- The study demonstrates how heterogeneous knowledge sources—including domain literature, expert insights, and caregiver perspectives—can be integrated using a structured ontology engineering methodology to represent complex caregiving dynamics.
- By defining well-structured concepts and relations, EDEM-CONNECTONTO enables ontology-guided annotation schemes and facilitates automated information extraction from dementia-related textual data.
- The ontology provides the semantic backbone for GraphRAG-based caregiver-support systems, enabling structured knowledge retrieval and explainable assistance for managing agitation-related behaviours in PwD.

A variety of methodologies for ontology development have been proposed in the literature, including the Agile Methodology for Ontology Development Abdelghany et al. (2019), the Software-Centric Innovative John et al. (2017), the ENTERPRISE methodology Uschold and Gruninger (1996), and the TOVE methodology Gruninger (1995). In Abdelghany et al. (2019), the Agile Methodology applies agile principles from software engineering to ontology modelling and consists of three iterative phases: pre-game, development, and post-game. The Software-Centric Innovative Methodology John et al. (2017) defines a structured workflow comprising planning, conceptualisation, development, implementation, and evaluation. Falbo’s Systematic Approach for Building Ontologies (SABiO) Aguiar and Souza (2024) guides through five main stages: defining purpose and requirements, capturing and formalising knowledge, designing the ontology, implementing it, and performing tests. Among ontology engineering methods, the most prominent methods in the health and biomedical domains are METHONTOLOGY Fernández-López et al. (1997) and the NeOn methodology Suárez-Figueroa et al. (2011). METHONTOLOGY adopts a knowledge-level perspective Pinto and Martins (2004) and prescribes a structured process involving specification, conceptualisation, formalisation, implementation, and maintenance. The NeOn methodology extends this by supporting dynamic ontology networks, the reuse of existing ontologies, and iterative development cycles suitable for large-scale, collaborative projects.

The remainder of this paper is structured as follows. Section 2 describes the methodology used to develop EDEM-CONNECTONTO, including the knowledge elicitation process and ontology engineering approach. Section 3 presents the resulting ontology and its evaluation. Section 4 discusses its applications and implications for caregiver-support systems and outlines the limitations of the proposed approach. Finally, Section 5 concludes the paper and highlights directions for future work.

## 2 Methods

**FIGURE 1:**
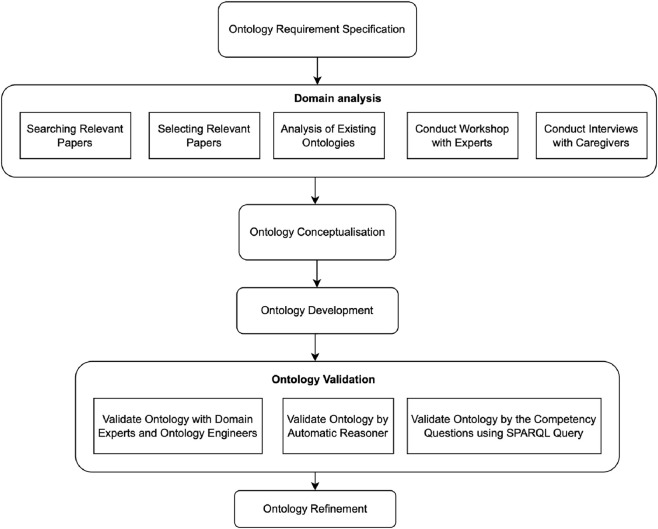
Schematic flow of the ontology development process. The development consists of two phases: (1) initial ontology development based on the collected domain knowledge, (2) further development of the ontology by integrating existing ontologies and new requirements, and then final validation of the ontology by domain experts, ontology engineers and automatic inference.

The EDEM-CONNECTONTO was developed to capture domain knowledge related to the various forms of agitation observed in PwD, as well as the dyadic relationship between PwD and their informal caregivers, addressing limitations identified in existing ontologies. The ontology also provides a structured representation of non-pharmacological interventions, enabling systematic modelling of strategies aimed at mitigating the bidirectional effects of agitation in PwD. The development process followed the NeOn methodology Suárez-Figueroa et al. (2011), a well-established framework for ontology engineering that has been widely applied in the health domain (Mazo et al., 2017; Berges et al., 2016). The NeOn methodology was selected because it supports the integration of heterogeneous knowledge sources and enables iterative ontology development. This flexibility is particularly important for modelling dementia-related agitation behaviours, which require combining domain-specific knowledge from heterogeneous sources. The methodology involves several key steps, including the specification of ontology requirements, conceptualisation of the domain, and implementation of the ontology in the Web Ontology Language (OWL). The development process (see Figure 1) was guided by a set of competency questions (CQs) designed to ensure that the ontology adequately captures the intended use cases and domain knowledge.

### 2.1 Ontology requirement specification

**FIGURE 2:**
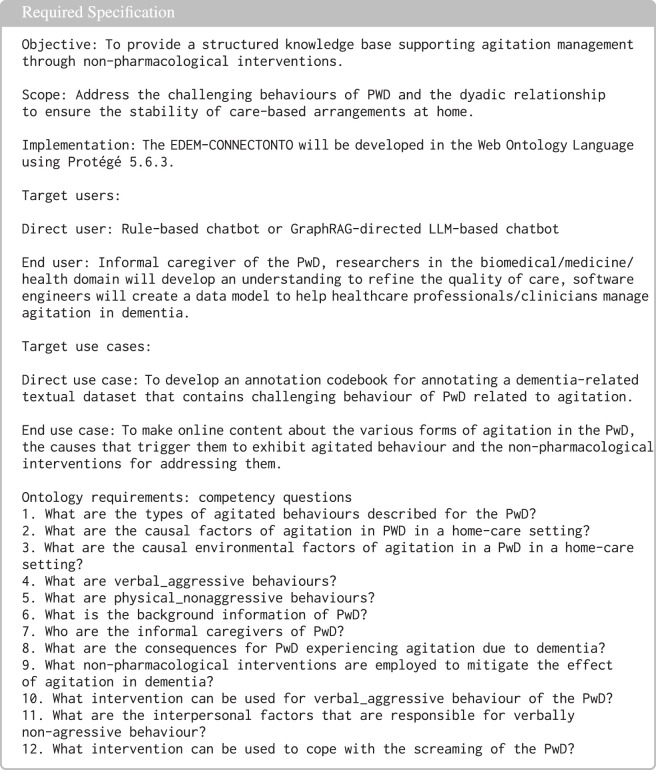
Ontology requirements specification for the EDEM-CONNECTONTO.

The requirements that the EDEM-CONNECTONTO needs to satisfy were determined by the stakeholders, including domain experts, caregivers, and computer scientists. The ontology requirements specification includes the information about the objective, the scope, the target group, the implementing tool, and the intended use cases of the ontology (see Figure 2) and specifies the CQ.

### 2.2 Domain analysis

To gather domain knowledge on agitation of PwD and on strategies for dealing with agitation, several domain analysis activities have been performed. They involved examining multiple use scenarios, identifying existing ontologies that could be partially reused into EDEM-CONNECTONTO, and analysing domain-specific challenges. The process also included a systematic review of relevant literature, the identification of domain knowledge through questionnaires and interviews conducted with domain experts, an expert workshop, and an evaluation of existing solutions in the field.

#### 2.2.1 Searching and selecting relevant resources

A comprehensive and unbiased literature review was conducted without discrimination based on race, sex, migration status, ethical background, or geographical origin of the studies. An extensive and systematic search of existing ontologies was performed using the keywords “agitation,” “dementia,” “dyadic relationship,” “dementia care,” and “non-pharmacological intervention.” Searches were conducted across major biomedical ontology repositories, including the National Center for Biomedical Ontology (NCBO) BioPortal Geller et al. (2018). Relevant knowledge was extracted from the DRANPTO ontology Zhang et al. (2020) based on these search terms. In addition, a systematic literature review was conducted in consultation with domain experts using the databases PubMed, Scopus, Google Scholar, MEDLINE, and Web of Science. To ensure comprehensive coverage, reference lists of eligible studies were also manually screened. The review process followed the Preferred Reporting Items for Systematic Reviews and Meta-Analyses guidelines (Moher et al., 2009). The search strategy includes the availability of full free-text and the selected vocabulary related to agitation, behavioural and psychological symptoms of being agitated, and non-pharmacological interventions. Titles and abstracts were initially screened, followed by full-text assessment after the removal of duplicate records. A total of 477 articles were identified in the initial search. After removing 62 duplicates, 415 articles remained. Of these, 408 articles had full-text availability. Studies classified as preprints, clinical intervention trials, poster papers, or abstract-only publications were excluded. This resulted in 95 articles eligible for in-depth review. These articles were thoroughly evaluated by the ontology engineer in consultation with domain experts. Following this process, 18 studies were selected. These studies were included in the review considering the following criteria: participants were aged 45 years or older, had a confirmed diagnosis of dementia, and exhibited agitation as defined by the Cohen–Mansfield Agitation Inventory model (Cohen-Mansfield, 1997). In addition, only studies focusing exclusively on non-pharmacological interventions were selected. Studies were excluded if they examined agitation associated with other neurodegenerative diseases or behaviours along with the pharmacological or clinical interventions, did not address dementia care, or investigated behavioural symptoms other than agitation. Furthermore, conference abstracts, posters, editorials, commentaries, dissertations, and other non-peer-reviewed materials were excluded from the review.

#### 2.2.2 Interviews with family-caregivers

**FIGURE 3:**
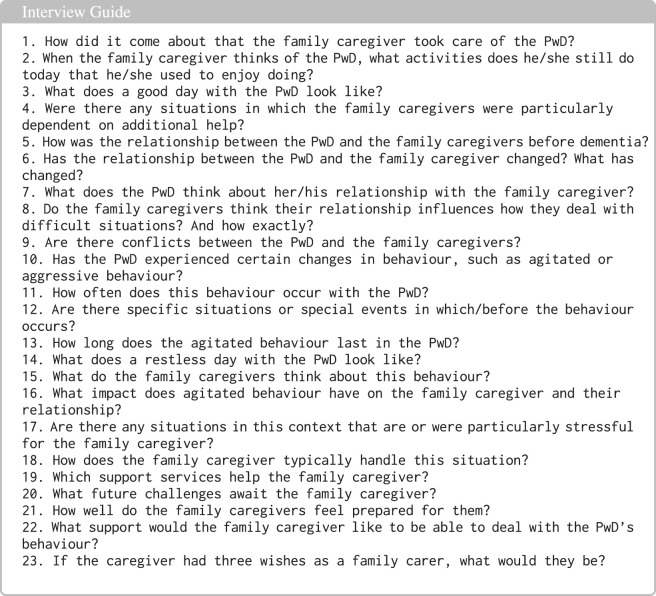
Interview questions.

Qualitative interviews were conducted with 12 family caregivers of PwD to validate the ontology concepts. The interviews, structured by an interview guide (see Figure 3), have been digitally recorded and analysed using content analysis. The guided interviews Kruse (2015) with relatives of PwD served to conceptualise the further development and validation of the first version of the EDEM-CONNECTONTO on agitation in PwD and stability of care in the home. According to the interview questions, the following aspects were addressed thematically in the guide: the care arrangement, causes and characteristics of agitation, strategies for coping with agitation, and the associated solution strategies. To this end, we used questions from interview guidelines already in use in this area within the institution and supplemented them with additional questions arising from the research interests of the project consortium. Concerning stability, due to the complexity Köhler et al. (2021) of the phenomenon, it was decided to focus on the concept of the dyadic relationship, which describes the relationship between PwD and their caregiving relatives. The focus of the interviews is on the importance of the dyadic relationship for dealing with agitation and on the quality of the relationship or changes in it over the course of the disease, as well as related aspects such as the motivation to seek support. The findings from the interviews with informal caregivers, together with insights from workshops conducted with nursing scientists, were analysed to identify key concepts, relationships, and caregiving contexts related to agitation in PwD. These identified concepts and relationships were subsequently integrated into the ontology model and used to refine the conceptual structure of EDEM-CONNECTONTO. In addition, the themes identified through qualitative analysis and further refined during the workshops were translated into ontology requirements and formalised as competency questions (CQs), which guided the ontology development process and were used to verify whether the ontology adequately represents the relevant domain knowledge and caregiving scenarios. Participants were recruited among informal caregivers who self-identified as primary caregivers of a PwD and were willing to share experiences of challenging care situations related to agitation and restlessness. Their lived experiences provided valuable insights into the realities and complexities of home-based dementia care.

#### 2.2.3 Workshops with experts

In an expert workshop, with 6 experts in counselling relatives of the PwD, the preliminary ontology has been presented and discussed. In terms of completeness, hints were given on the linguistic adaptation of terms to the target group. Fourteen concepts mainly related to the causes of agitation were also added and prioritised to complete the ontology.

#### 2.2.4 Integrating existing ontologies

The EDEM-CONNECTONTO reused concepts from the DRANPTO ontology Zhang et al. (2020), which focuses on the non-pharmacological management of agitation in dementia. 65 concepts were mapped from DRANPTO, describing a range of agitated behaviours. Although DRANPTO includes behavioural manifestations, causative factors, and non-pharmacological interventions, it does not fully meet the modelling requirements of EDEM-CONNECTONTO. In particular, its causal layer lacks the granularity needed for home-care contexts and does not capture person-specific background factors. For this reason, only the behavioural concepts were reused. DRANPTO’s causative categories are broad and not agitation-specific, making them unsuitable for integration without introducing semantic overlap or inconsistencies. Instead, EDEM-CONNECTONTO introduces a new, evidence-based causal model grounded in Cohen–Mansfield Agitation Inventory (CMAI), Innovative dementia orientated Assessment system (IdA-assessment), and insights from expert workshops. Besides, five concepts, four related to the technical affinity of the user and one to the medical history, were extracted from the SiNDem ontology Yordanova et al. (2017): “Technical affinity,” “Type_1_user,” “Type_2_user,” “Type_3_user,” “Medical history.”

### 2.3 The process of knowledge conceptualization

In the conceptualisation phase, domain experts identified relationships between relevant concepts. The initial ontology was implemented in OWL W3C (2013) and then automatically validated by logical inference. As the initial ontology is relatively general and incomplete, the ontology was further developed in the second phase, which addressed the further development of the ontology on new knowledge resulting from user questionnaires, interviews, and expert knowledge not identified in the first phase. The newly discovered knowledge was then integrated into the initial ontology. The last step of the second phase of the development process is the further model implementation and validation by logical inference in the Protégé 5.6.3 software Musen (2015). Parts of existing ontologies were additionally integrated into the ontology to ensure reusability, and the ontology model was validated by the ontology engineer and the domain experts, who checked for completeness and clarity.

The conceptual model was developed as follows. At first, the important terms representing the nature of agitated behaviour and the causes of being agitated were elicited from the selected knowledge source and coded in the ontology development tool. These terms were defined as nouns and verbs. Here, we identified nouns as concepts and individuals, and then added concepts that include metadata information. Two types of metadata were incorporated into the ontology. The first type captures metadata at the ontology level, such as licensing and general description of EDEM-CONNECTONTO. The second type pertains to individual ontology elements and includes annotations such as class definition, labels, and source references. The class hierarchy was constructed following the subsumption relationship (i.e., is-a-superclass-of). For instance, the terms “Verbal_aggressive” and “Verbal_nonaggressive” are the sub-concepts of “Agitation”. The elicited verbs were defined as object properties for determining inter-relationships between the concepts within the domain and range. For instance, the verb “exhibits” was elicited as an object property for defining the relationships between the classes “People with dementia” and “Agitation”, where the class “People with dementia” is denoted as domain, and the class “Agitation” is denoted as the range of the object property.

### 2.4 EDEM-CONNECTONTO development

The concepts in an ontology are defined as the class of entities, whereas the instances are the concrete situation-specific “things” that represent the concepts. The ontology is implemented in OWL Antoniou and van Harmelen (2009) using the Protégé 5.6.3 software Musen (2015). OWL is a semantic web language developed to introduce information about the concepts, individuals, and relations between the concepts (Musen, 2015). During the ontology development phase, a top-down hierarchical approach was adopted to ensure conceptual consistency. This approach begins by defining high-level domain concepts and progressively refining them into more specific subclasses and relationships. Such a hierarchical modelling strategy helps maintain logical consistency across the ontology structure and facilitates reuse and extensibility, as new concepts and relationships can be systematically integrated into the existing conceptual hierarchy (Zhang et al., 2020, 2024; Gangemi et al., 2005; Wilson et al., 2023). Starting from top-level domain concepts, such as Person and Agitation, and refining them into more specific subclasses, such as Caregiver and Verbal_aggressive, allowed EDEM-CONNECTONTO to maintain a coherent structure while progressively incorporating more detailed behavioural and contextual knowledge.

### 2.5 EDEM-CONNECTONTO evaluation

A variety of ontology evaluation methods have been developed to ensure the correctness, consistency, and suitability of ontological models. Tool-based evaluation approaches commonly employ semantic reasoners such as HermiT Glimm et al. (2014), Pellet Sirin et al. (2007) to test logical consistency and detect modelling conflicts Abburu (2012). In addition, the OntOlogy Pitfall Scanner (OOPS!) Poveda-Villalón et al. (2014) is widely used to identify common modelling errors and structural pitfalls. Beyond automated tools, ontologies are also assessed through expert-based evaluation, competency question testing, and quality criteria frameworks, which togetherhelp verify whether the ontology faithfully represents the domain and supports its intended use (Raad and Cruz, 2015). The EDEM-CONNECTONTO was assessed for consistency with the HermiT and Pallet reasoners as well as with the automatic logical reasoner OOPS! and through SPARQL queries. Finally, the EDEM-CONNECTONTO was evaluated by the standards of the biomedical ontology in terms of accuracy, clarity, completeness, conciseness, and consistency (Amith et al., 2018). Section 4 also provides a list of machine learning related tasks, to which the ontology has already been applied.

## 3 Results

The EDEM-CONNECTONTO was evaluated using automated logical reasoners to ensure the consistency and coherence of the ontology. Specifically, the Pellet Sirin et al. (2007) and HermiT Glimm et al. (2014) reasoners were employed to detect logical inconsistencies and identify redundant relationships within the model. In addition, the OntOlogy Pitfall Scanner (OOPS!) Poveda-Villalón et al. (2014) was applied to assess potential modelling issues. OOPS! is a web-based ontology evaluation tool that analyses ontologies against 41 well-established pitfalls. These pitfalls were derived from systematic analyses of existing ontologies, and were further validated through user feedback and an empirical study involving 969 ontologies Poveda Villalón (2016). In addition, EDEM-CONNECTONTO was assessed to check whether it could answer the CQ using the SPARQL query language. SPARQL Seaborne and Prud’hommeaux (2008) is a semantic query language designed for querying and manipulating data represented in RDF-based ontologies. The EDEM-CONNECTONTO was evaluated by the standards of the biomedical ontology in terms of accuracy, clarity, completeness, conciseness, and consistency (Amith et al., 2018). Two dementia care experts, an experienced professor of the Nursing Department at the University of Witten and an experienced manager of the eDEM-Connect project, were asked to manually assess the EDEM-CONNECTONTO in terms of accuracy, clarity, and completeness. Two annotators were also requested to annotate dementia-related data using an annotation codebook developed from the EDEM-CONNECTONTO. The annotated corpus was employed to assess whether the concepts defined in the EDEM-CONNECTONTO are recognisable by the annotator in dementia-related informal texts.

### 3.1 Results from literature review

In EDEM-CONNECTONTO, concepts were derived from 18 research articles, covering the target domain related to PwD. The concepts and their origin can be seen in Table 1. The details about the literature review can be found in the Supplementary Appendix.

**TABLE 1:** Concepts of EDEM-CONNECTONTO, extracted from literature.

References	Concepts elicited
Yordanova et al. (2017)	Five concepts: “Technical affinity,” “Type_1_user,” “Type_2_user,” “Type_3_user,” “Medical history” were extracted from this study
Bartholomeyczik and Halek (2010)	All the concepts of “Independence in activities of daily living,” “Lifestyle before dementia” and “Mood and emotion” are extracted from this study. In total, 20 concepts extracted from this study
Bartholomeyczik and Halek (2010)	34 sub-concepts of the upper-most concept “Topography” are extracted from this study
Cohen-Mansfield (1997), Baillon et al. (2004)	38 sub-concepts of “Physical_aggressive,” “Physical_nonaggressive”
Cohen-Mansfield and Libin (2005), Cohen-Mansfield (2008)	“Verbal_aggressive,” “Verbal_nonaggressive” were extracted
Alexopoulos et al. (2007), DEMENTIA (2007)	6 sub-concepts of “Strong emotion” were extracted from this study
Laybourne (2019)	32 sub-concepts of “Intervention” were extracted from this study
Kales et al. (2015)	26 sub-concepts of “Factors related to the people with dementia,” 27 sub-concepts of “Factors related to the people with dementia,” 6 sub-concepts of “Environmental factors,” and 18 sub-concepts of “Caregiver factors” were extracted from this study
Cohen-Mansfield and Parpura-Gill (2007)	The Treatment Routes for Exploring Agitation (TREA) approach is applied in this study. “Sleep disorder” and “Pain of the person with dementia” were extracted from this study
Kolanowski et al. (2011)	“Temperature,” “Light level” and “Noise level” were extracted from this study
Lawlor and Sunderland (1994)	“Change in routine,” extracted from this study
Rhodes-Kropf et al. (2011)	“Phone call frequency,” “Visitor visit frequency” were extracted from this study
Kales et al. (2015)	8 concepts of “Consequences” were extracted from this study
Kong (2005)	4 concepts of “Consequences” were extracted
Ward-Griffin et al. (2007)	“Family-carer” concept, extracted from this study
Köhler et al. (2021)	“Job/employment” and “Living situation” concepts extracted from this study
Thomas and Harden (2008)	​
Braun et al. (2009)	“Gender” concepts, extracted from this study

### 3.2 The resulting EDEM-CONNECTONTO

The EDEM-CONNECTONTO has 252 concepts, 16 relations (see Table 2), and 241 individuals based on the agitation of PwD, social and physical environment, family members, causes, consequences, health issues of the PwD, and intervention strategies. The 7 higher-most concepts in the EDEM-CONNECTONTO are “Person”, “Agitation”, “Causes”, “Consequences”, “Interventions”, “Profile” and “Topography” (see Table 3). The higher-most concepts were structured across five levels of granularity. Among these, the concepts “Causes” and “Profile” exhibited the greatest depth, each extending to the fifth level of the hierarchy, reflecting their complexity and central role within the ontology. As it is sensible to generate deeper knowledge and further specify the problem with additional diagnostic questions as child concepts, we added concepts in a top-down hierarchical way. For instance, the “Agitation” concept has the sub-concept “Verbal_aggressive”, which is then divided into sub-subconcepts “screaming” and “exhibits” relation is created between the concept “People with dementia” and “Agitation”. The last step was creating individuals of the concepts in the hierarchy. 241 German translations of the concepts were added as individuals in the EDEM-CONNECTONTO. All the concepts of the EDEM-CONNECTONTO are available with metadata information in BioPortal.^2^ Each concept is defined as a class including a label, which is a human-readable class name, comment, abbreviation, class-mapping if applicable, structural metadata such as Internationalised Resource Identifier (IRI), direct superclass, direct subclasses, and relationships with other classes if applicable. Details about the ontology and its structure could be found in the Supplementary Appendix.

**TABLE 2:** Relationships between the concepts and their definitions.

Relationship	Definition
Is-a	Denotes that the concept is a sub-concept of another concept
Exhibits	Indicates relation between “PwD” and “Agitation” concept
Triggers	Indicates relation between the “Causes” and “Agitation” concepts
Allows	Indicates relation between “Acceptance” (e.g., permit to show agitation) and “Agitation”
Leads	Indicates relation between the “Agitation” and “Consequences” concept
Manages	Indicates relation between “Attendee” and “Agitation” concept
Occurs	Indicates relation between “Agitation” and “Situation” (e.g., Dressing, Food_intake) concept
Prevents	Indicates relation between “Prevention” (e.g., Maintain routines) and “Agitation” concept
Reduces	Indicates relation between “Acceptance” and “Consequences” concept
Resolves	Indicates relation between “Problem_solving” (e.g., Screening for medical causes) and “Consequences” concept
Has	Indicates relation between “Person” and “Technical affinity” concept
Hascaregiverprofile	Indicates relation between “Caregiver” and “Profile of caregiver” concept
Hasfrequency	Indicates relation between “Agitation” and “Frequency” (e.g., daily, weekly) concept
Hasprofile	Indicates relation between “People with dementia” and “Profile of people with dementia” (e.g., occupation) concept
Haslocation	Indicates relation between “Agitation” and “Places” concept
Hastemporalpattern	Indicates relation between “Agitation” aand “Duration” (short-term, Continuous) concept

**TABLE 3:** Hierarchical distribution of concepts in EDEM-CONNECTONTO with the upper-most concept and the number of concepts at all levels.

Upper-most concepts	1st level	2nd level	3rd level	4th level	5th level
Agitation	1	5	38	0	0
Causes	1	3	41	19	11
Consequences	1	9	3	0	0
Interventions	1	5	26	0	0
Person	1	2	4	0	0
Profile	1	3	19	19	4
Topography	1	5	29	0	0

### 3.3 Assessing the quality of EDEM-CONNECTONTO

EDEM-CONNECTONTO was assessed with respect to consistency, clarity, completeness, and accuracy following the biomedical ontology standards Amith et al. (2018). Consistency assessment ensures that the ontology does not contain or permit any logical contradictions. Accordingly, consistency was evaluated using the Pellet Sirin et al. (2007) and HermiT Glimm et al. (2014) reasoners. The results of this evaluation confirmed that the EDEM-CONNECTONTO is logically consistent and free of conflicting axioms. Clarity ensures that the ontology should be unambiguous, with well-defined meanings for all concepts and relationships. Clarity of the EDEM-CONNECTONTO is accomplished by assigning a specific label with a relevant explanation to the concepts individually using “rdfs: label” “rdfs: comment.” It also ensures that the ontology can communicate effectively with readers, providing a relevant explanation of the concepts. Regarding assessing accuracy, more than 100 warnings were discovered by domain experts and the Ontology pitfall scanners. These warnings and potential pitfalls were systematically reviewed and resolved through iterative discussions between the ontology engineer and domain experts, leading to successive refinements of the ontology. According to the biomedical ontology standards, EDEM-CONNECTONTO is assessed with respect to measuring completeness. Completeness basically measures the ontology’s domain knowledge coverage (Amith et al., 2018). Two domain experts and an experienced manager of the eDEM-CONNECT project evaluated the completeness of EDEM-CONNECTONTO manually. Their revision on EDEM-CONNECTONTO is viewed as a genuine assessment. Based on their suggestions, we added four new concepts as the ontology’s sub-subconcepts of the “Interventions” concept. They were: “Self-care: taking time for yourself,” “Looking for distractions,” “Safety for PwD: provide a safe environment” and “Safety for relatives: leave the room, leave PwD alone” as a subclass of “Reduction of negative consequences.” In addition, following the domain expert’s recommendation about redundancy, the term “Talking about feelings and experiences of the PwD” was removed from the subclass of “Reduction of negative consequences.” The concepts “distress,” “reduced income from employment” and “depression” were also removed from the subconcepts of the “Consequences” concept. The domain experts and the project manager then evaluated the modified ontology. They verified its accuracy and completeness without additional revision.

### 3.4 Assessing EDEM-CONNECTONTO by competency questions using SPARQL queries

The capability of the EDEM-CONNECTONTO to answer the CQ was evaluated using SPARQL query (Seaborne and Prud’hommeaux, 2008). By using SPARQL query language, we retrieved relevant data from the EDEM-CONNECTONTO using the 12 CQ. The retrieved data were checked to see if the EDEM-CONNECTONTO could deliver the correct response for each CQ.

The retrieved outputs for each CQ using SPARQL query are presented in Table 4.

**TABLE 4:** Retrieved outputs from the EDEM-CONNECTONTO based on the competency questions.

CQ	Retrieved outputs
1. What are the types of agitated behaviours described for the people with dementia?	“Strong_emotion”, “Verbal_aggressive”, “Verbal_nonaggressive”, “Physical_aggressive”, and “Physical_nonaggressive”
2. What are the causal factors of agitation in people with dementia?	Environmental factors”, “Interpersonal factors”, and “Factors related to the person with dementia”
3. What are the causal environmental factors of agitation in person with dementia?	“Lack of activity”, “Lack of established routines”, “Safety issues”, “Change in routines”, “Light level”, and “Noise level”
4. What are verbal aggressive behaviours?	“Screaming”, “Cursing”, “Safety issues”, and “Making verbal sexual advances”
5. What are physical nonaggressive behaviours ?	“Aimless wandering”, “Pacing”, “Hyper activity”, “Hiding things”, “Intentional falling”, “Handling things inappropriately”, “Eating inappropriate substances”and “Performing repetitive mannerism”
6. What is the background information of people with dementia?	“Mood and emotion”, “Personality”, “Lifestyle before dementia”, “Age of the person with dementia”, “Preferences”, and “Resources”
7. Who are the informal caregivers of people with dementia?	“Family_carer”, “Neighbours”, “Friends”, and “Further_family_carer
8. What are the consequences for PwD experiencing agitation due to dementia?	“Anorexia_weight_loss”, “Decreased_quality_of_life”, “Dehydration”, “Disability”, “Hospital_admission”, “Higher_incidences_of_falling”
​	“Institutionalisation”, “Interpersonal_relationship” and “Poor_caregiver_outcomes”
9. What non-pharmacological interventions are employed to mitigate the effect of agitation in dementia?	“Maintain routines”, “provide for social contacts”, “Promote wellbeing”, “Improve the quality of relationship between the PwD and the relative” and “Identify unmet needs and meet them when possibles”
10. What intervention can be used for verbal_aggressive behaviour of the people with dementia?	“Distract”, “Screening for physical/medical causes”, “Environmental design”, “Recognize and avoid triggers” and “Screening for physical/medical causes”
11. What are the interpersonal factors that are responsible for verbally non-agressive behavior?	“Lack of caring skills,” “Language of the caregiver,” “verbal interaction” and “Low mental capabilities”
12. What intervention can be used to cope with the screaming of the people with dementia?	“Communication: controlling the emotional tone of your own language” and “Distract”

### 3.5 Assessing EDEM-CONNECTONTO using OntOlogy pitfall scanner (OOPS!)

EDEM-CONNECTONTO is assessed by OOPS! OOPS! detected pitfalls on three levels according to impact on the ontology: critical, important, and minor. Critical pitfall refers to the most crucial pitfall that needs to be corrected. Otherwise, it could affect the ontology in terms of consistency, reasoning, and applicability. Using OOPS!, no critical pitfall was reported. Two important and twelve minor pitfalls were detected, resolved by discussing with the domain experts and the ontology engineer. One of the two important pitfalls was that two pairs of classes might be equivalent, yet not explicitly declared in EDEM-CONNECTONTO. They were “Hunger” vs. “Thirst’, ‘Situation’ vs. “Places”. To address this problem, further detailed discussions were held with domain experts and the ontology engineers. Both experts and the ontology engineer did not agree with the OOPS! scanner. For the first pair of classes, “Hunger” vs. “Thirst”, they were not equivalent concepts because “Hunger” is an uneasy sensation occasioned by the lack of food” Merriam-Webster (2019a); whereas “Thirst” is “a sensation of dryness in the mouth and throat associated with a desire for liquid” Merriam-Webster (2019b) according to Merriam Webster Dictionary. Therefore, the corresponding descriptions of these two concepts were added as “rdfs: comment” in EDEM-CONNECTONTO. Domain experts also could not agree with the second reported pair of classes, “Situation” vs. “Places,” as both concepts convey different meanings semantically. In EDEM-CONNECTONTO, the concept “Situation” refers to the different circumstances, such as going for shopping, where agitation occurs over a period of time, whereas the concept “Places” refers to the places, such as the basement or bathroom, where agitation occurs over a particular period of time. Another important pitfall was that the ontology metadata omitted information about the license that applies to EDEM-CONNECTONTO. To fix it, the license information “CC BY-NC-SA 4.0” was included as an annotation property “dcterms: license” in EDEM-CONNECTONTO. OOPS! identified the P08 pitfall (missing annotations), which occurs when ontology elements are created without human-readable annotations. In our case, OOPS! reported missing “rdfs:label” annotations for the data properties: hasTranslation and hasSynonym. We therefore added appropriate “rdfs: label” values for these properties in Protégé. OOPS! also flagged P08 for several classes lacking “rdfs:comment.” Consequently, we added missing “rdfs:comment” annotations for the classes: Attendees, Duration, Problem_solving, Looking_for_distraction, Anticipate, and Disability. Furthermore, OOPS! reported the P13 pitfall, which indicates that inverse relationships are not explicitly declared. This affected the object properties allows, triggers, reduces, resolves, and prevents. To address this, we explicitly defined the corresponding inverse object properties for each of these relations using Protégé.

## 4 Discussion

### 4.1 Applications of EDEM-CONNECTONTO

#### 4.1.1 Overview of applications

**FIGURE 4:**
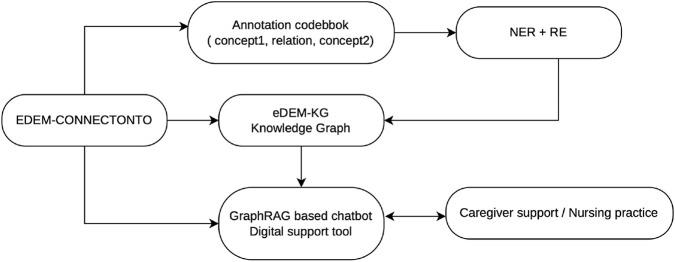
Ontology-driven workflow for dementia caregiver support. EDEM-CONNECTONTO provides the semantic foundation for ontology-guided annotation, information extraction using named entity recognition and relation extraction, populating the knowledge graph and GraphRAG-based chatbot.

The applications supported by EDEM-CONNECTONTO follow a coherent utilization workflow (see Figure 4). First, the ontology provides a formal semantic framework that guides data annotation and information extraction from caregiver narratives through NER and RE pipelines. The extracted entities and relations are subsequently used to populate the ontology-driven knowledge graph (eDEM-KG), which organizes agitation-related events, triggers, and caregiving responses in a machine-interpretable structure which can be used in GraphRAG approaches. This allows, on the one hand, to use EDEM-CONNECTONTO itself as a knowledge base in GraphRAG-based chatbot systems or to use it as a basis for the automated construction of knowledge graphs, which are then applied to GraphRAG approaches for supporting the family caregivers of PwD.

#### 4.1.2 Dementia dataset

An annotation scheme was proposed following the 8 most general concepts and relations from the EDEM-CONNECTONTO for annotating textual data from online dementia forums.^3^ “Agitation”, “Verbal_aggressive”, “Verbal_nonaggressive”, “Physical_aggressive”, “Physical_nonaggressive”, “Causes”, “PwD”, “Family-carer” are the labels used for developing a dementia dataset (see details in Suravee et al. (2022)). The annotated dementia dataset, Informal Dementia Forum Corpus (IDFC), consisted of 1641 entities based on the above-mentioned labels (see details in Suravee et al. (2022)). Two annotators were employed to label instances independently of the entities (concepts) and the relationships in the dataset. We used Cohen’s kappa score, incorporated from Landis and Koch (1977) as a measure of inter-rater reliability to evaluate the quality of the annotated data. In our case, the inter-rater agreement for the entities is κ = 0.49, which shows that we have a moderate score. We observed that the annotators mainly annotated entities containing noun phrases (e.g., father, he) based on “PwD” and “Family-carer” labels compared to the labels describing types of agitation of PwD (e.g., verbal_nonaggressive, physical_aggressive) (see Suravee et al., 2022). We found that the domain of PwD is difficult to conceptualize. Our domain experts and the annotators struggled with identifying the concepts and relationships that define the domain. Nonetheless, the outcomes demonstrated that both annotators could observe the labels from the concepts in EDEM-CONNECTONTO with moderate agreement.

#### 4.1.3 Named entity recognition (NER)

It enables the automated extraction of structured, domain-specific information from unstructured textual data. EDEM-CONNECTONTO was integrated into the experimental NER pipeline, where its concepts were used to annotate the IDFC dataset mentioned above (Suravee et al., 2022; Suravee et al., 2025a). IDFC was subsequently employed to train an NER model for identifying ontology-driven entities in online dementia-related texts. The concepts derived from EDEM-CONNECTONTO were used to train a bidirectional long short-term memory (Bi-LSTM) model with the conditional random field (CRF) classifier for ontology-driven entity recognition. The experimental results demonstrated that the model achieved a macro F1-score of 0.58 in identifying rare, ontology-derived categories such as agitation, causal factors, and sub-classes of “Agitation” such as “Verbal_aggressive” within informal, context-rich dementia forum texts Suravee et al. (2025a) (see Table 5). These findings highlight the utility of EDEM-CONNECTONTO in supporting dataset construction and enabling supervised learning for an ontology-driven entity recognition task.

**TABLE 5:** F1 scores on the dementia dataset using traditional neural networks for each entity type: PwD, Causes, Family-carer, Agitation, Verbal_aggressive, Verbal_nonaggressive, Physical_aggressive, Physical_nonaggressive and the relationship entity: exhibits, triggers.

Models/NE labels	Bi-LSTM-CRF
PwD	0.91
Family-carer	0.87
Causes	0.53
Agitation	1.0
Verbal-aggressive	0.16
Verbal-nonaggressive	0.71
Physical-aggressive	0.10
Physical-nonaggressive	0.38
Exhibits	0.98
Triggers	0.98

#### 4.1.4 Relation extraction (RE)

It aims to identify semantic relationships between named entities. In our case, it was leveraged to automatically identify and annotate semantic relations between ontology-driven entities in the IDFC dataset (Suravee et al., 2025b). EDEM-CONNECTONTO was employed to define the target domain and guide the development of an experimental RE pipeline. Within this framework, the ontology-defined relationships “exhibits” and “triggers” were used as relation labels to train a Bi-LSTM model with a CRF classifier. The trained model achieved strong performance, attaining an overall F1-score of 0.81, with an F1-score of 0.98 for the identification of the “exhibits” and “triggers” relations (see Table 5). The output of the RE process consists of structured relational annotations that can be directly integrated into downstream knowledge bases. These results demonstrate the effectiveness of EDEM-CONNECTONTO in supporting automated relation extraction and highlight its potential for transforming unstructured dementia-related text into richly annotated, machine-interpretable resources without manual intervention.

#### 4.1.5 GraphRAG-based chatbot

**FIGURE 5:**
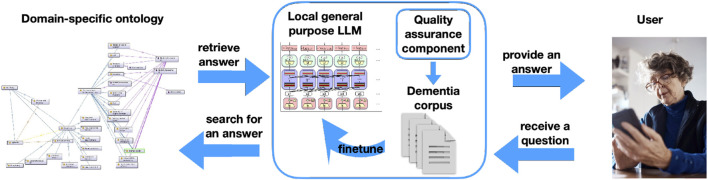
Overview of developing a GraphRAG-based chatbot using EDEM-CONNECTONTO and automatic knowledge extraction from real-world domain-specific forum texts (Suravee et al., 2026).

EDEM-CONNECTONTO was used as a knowledge base for a privacy-preserving support system that operates entirely on a local home environment (Suravee et al., 2026). By running locally, the system avoids reliance on external servers, thereby ensuring that sensitive user data remain private while providing continuous, accessible support for informal dementia caregivers. A large language model (LLM) serves as an intermediary between the user and the ontology, retrieving relevant knowledge from EDEM-CONNECTONTO and generating user-friendly responses based on this structured information (see Figure 5).

To address privacy and security concerns, particularly critical in dementia-related applications, a locally deployable LLM architecture was adopted. Two models were evaluated: Meta’s quantised LLaMA-3.3-70B and the lighter LLaMA-3-8B model (Grattafiori et al., 2024). The experimental evaluation was conducted using 25 question–answer pairs collected from IDFC. For each question, responses were generated under three configurations: (i) a LLM without EDEM-CONNECTONTO grounding, (ii) an ontology-guided LLM retrieving concepts and relations from EDEM-CONNECTONTO (Onto_LLM), and (iii) an ontology-guided LLM restricted to 32 intervention-related concepts (Inter_LLM) (Suravee et al., 2026). EDEM-CONNECTONTO was parsed using RDFLib, and all concepts were indexed using their labels and descriptive annotations. To evaluate response quality, semantic similarity was computed across five comparison settings: user response vs. plain LLM output, user response vs. Onto_LLM output, user response vs. Inter_LLM output, plain LLM vs. Onto_LLM, and plain LLM vs. Inter_LLM. Sentence embeddings were generated using the pretrained all-MiniLM-L6-v2 model,^4^ and cosine similarity was used as the evaluation metric (Suravee et al., 2026).

As shown in Table 6, the plain LLM achieved the highest similarity to user responses (0.62), reflecting the informal and narrative style commonly found in caregiver forums. However, ontology-guided responses remained semantically close to the plain LLM outputs, with similarity scores of 0.74 for both LLaMA-70B and LLaMA-8B models. This indicates that ontology grounding preserves the natural language characteristics of LLM outputs while introducing structured domain knowledge. Notably, the intervention-restricted configuration achieved the highest similarity between ontology-guided and plain LLM responses (0.72–0.75), suggesting that focusing on intervention-specific knowledge aligns particularly well with caregiver reasoning patterns. When comparing ontology-guided responses to user answers, the locally deployed LLaMA-8B model achieved comparable performance to the entire EDEM-CONNECTONTO configuration, demonstrating the feasibility of lightweight, privacy-preserving deployment for real-world dementia support applications.

**TABLE 6:** Cosine similarity scores for plain, ontology-guided, and intervention-guided LLM responses compared to user answers.

Models	User vs. LLM	User vs. Onto_LLM	User vs. Inter._LLM	Onto_LLM vs. LLM	Inter._LLM vs. LLM
Llama-70B	**0.62**	**0.56**	**0.58**	**0.74**	0.67
Llama-8B (local)	**0.62**	**0.56**	0.55	**0.74**	**0.75**

Cosine similarity measures the cosine of the angle between two vector representations, defined as the normalized dot product, where higher values indicate greater similarity.

#### 4.1.6 eDEM-KG: an ontology-driven knowledge graph for dementia-related agitation events

We developed a knowledge graph (KG), termed eDEM-KG, comprising 122 ontology classes, 6 object properties, and 58 individuals. The conceptual structure of the KG was directly derived from EDEM-CONNECTONTO, while instances and relationships were populated using manually annotated entities and relations from a BRAT-annotated IDFC (Suravee et al., 2022; Suravee et al., 2025b). This dataset contains ontology-driven annotations extracted from the real-world dementia forum texts. This resultant kG^5^ illustrates how unstructured dementia-related text can be transformed into structured semantic knowledge through ontology-driven extraction and linking of entities and relations. While this implementation uses manually curated annotations to maintain high reliability, the population workflow is transferable to entities and relations automatically identified by NER and RE models, supporting scalable knowledge graph generation.

#### 4.1.7 Nursing science

The EDEM-CONNECTONTO represents the first formalised attempt to capture the complexity of knowledge associated with agitation in PwD. This contribution is particularly significant, as nursing practice has long faced challenges of verbalising professional practice and making its complexity visible. The nursing diagnoses (NANDA) Kamitsuru et al. (2022) and taxonomies such as NIC Dochterman and Bulechek (2004) and NOC Moorhead et al. (2023) already exist, but they do not come close to reflecting the complexity of individual phenomena such as agitation. Overall, the development of the ontology has resulted in an exemplary representation of nursing phenomena, which can be used for various (technical) applications. It has been shown that it is possible to develop an ontology for nursing phenomena and that the methodological approach can therefore also be transferred to other topics in nursing care. For nursing science, the ontology provides a structure for developing assessments and nursing documentation, for providing knowledge to those involved in the care of people with dementia (learning materials), for analysing nursing data (supporting decision-making in the nursing process) and for nursing research (coding system). By representing the complexity of knowledge relevant to the care of this group of people, the ontology can contribute to the nursing science discussion about the competence requirements for caregivers with different levels of qualification.

### 4.2 Implications for nursing practice

From a nursing practice perspective, informal caregivers play a crucial role in providing day-to-day care for PwD, particularly in home-based care settings. However, caring for PwD over an extended period can place considerable emotional and psychological stress on caregivers, especially when dealing with challenging behaviours such as agitation. In many cases, informal caregivers effectively perform nursing-related tasks without having formal clinical training. By integrating EDEM-CONNECTONTO into digital support systems, such as ontology-driven chatbots, caregivers can access structured, dementia-specific knowledge and guidance on non-pharmacological intervention strategies. This structured knowledge can assist caregivers in understanding the triggers and characteristics of agitation, selecting appropriate coping strategies, and managing care situations more confidently. As a result, ontology-driven digital tools have the potential to support caregiver decision-making, improve the management of agitation-related behaviours, and reduce the overall caregiving burden.

### 4.3 Limitations of EDEM-CONNECTONTO

The development of EDEM-CONNECTONTO presented several methodological and practical challenges. The first major challenge concerned the identification of relevant knowledge sources within the dementia domain. In particular, extracting comprehensive and reliable information related to agitation types, non-pharmacological interventions, family caregivers, and the socio-economic context of PwD proved difficult due to the absence of a single, standardized resource that consolidates evidence-based knowledge across these dimensions. Consequently, relevant concepts had to be identified from a wide range of heterogeneous literature sources. During this process, domain experts reported difficulties in consistently defining and conceptualising the identified knowledge. A second major challenge involved selecting an appropriate and reliable ontology evaluation strategy. Ontology assessment remains an open research problem, as no single standardized method exists for evaluating ontology quality. Existing approaches include gold-standard comparison, domain expert–based evaluation, tool-supported validation, CQ–based evaluation, and quality-criteria-driven assessment (Amith et al., 2018). To address this challenge, a multi-faceted evaluation strategy was adopted for EDEM-CONNECTONTO. Specifically, the ontology was assessed through (i) automated reasoning to detect logical inconsistencies, (ii) CQ validation using SPARQL queries, (iii) evaluation against ontology quality criteria such as accuracy and clarity, and (iv) expert-based assessment. The combination of these complementary approaches provided a robust and reliable evaluation framework.

Another significant challenge was the involvement of domain experts in the evaluation process. Domain expert–based assessment requires careful manual inspection of ontology concepts and relationships, making it both time-consuming and resource-intensive. Despite these constraints, two domain experts and the eDEM-CONNECT project manager conducted a detailed review of the ontology, focusing on its accuracy, clarity, and completeness. In addition, two expert workshops were organised to discuss the conceptual structure, validate relationships, and refine terminology, ensuring that the ontology accurately reflected domain knowledge and practical caregiving realities. Despite these challenges, the development of EDEM-CONNECTONTO demonstrates strong potential for future applications. In particular, the EDEM-CONNECTONTO enables the transformation of dementia-related textual data into machine-interpretable representations through semantic annotation.

Although caregivers were actively involved throughout the development process, their participation in certain foundational design decisions was constrained due to limitations imposed during the COVID-19 pandemic. Furthermore, the scope of EDEM-CONNECTONTO is intentionally limited to non-clinical intervention strategies for managing agitation, as extensive clinical data addressing pharmacological aspects are already available in existing resources.

The evaluation of the ontology with respect to its applications has shown that the ontology has great potential for improving the quality and reliability of intelligent interactive support systems for the domain of dementia. The limitations faced in this respect are as follows: 1) the ontology-based annotation scheme, when applied to the IDFC corpus, shows a class imbalance, which is later also reflected in the ability of NER and RE approaches to extract information from informal text. This indicates that a dataset with a better class representation in required in order to annotate and train models with high performance according to the ontology-based annotation scheme; 2) the application of the ontology as a knowledge base in a GraphRAG approach is very promising, however a thorough evaluation in terms of performance as well as perceived usability is necessary to be able to make conclusive statements. This, however, opens a venue for practically applying the proposed approach and identifying future development perspectives.

Despite these limitations, the ontology provides an extensible foundation for supporting caregiver education, decision-making, and future intelligent applications in dementia care.

## 5 Conclusion and future work

This study introduced EDEM-CONNECTONTO, an ontology comprising 252 concepts designed to support multiple aspects of care for people with dementia (PwD). The ontology was developed following the NeOn methodology and is directly integrated into different tasks related to digital support systems. The EDEM-CONNECTONTO was developed in accordance with established biomedical ontology quality criteria, including accuracy, clarity, completeness, conciseness, and logical consistency. Both domain experts and ontology engineers were involved throughout the development process, ensuring that the ontology integrates scientific evidence alongside practical, experience-based knowledge. A distinguishing feature of EDEM-CONNECTONTO is its strong focus on family caregivers and the dyadic relationship between caregivers and PwD. This is reflected in the detailed modelling of agitation-related behaviours, their underlying causes, and corresponding non-pharmacological intervention strategies. By structuring this knowledge in a machine-interpretable form, the ontology provides a semantic foundation for ontology-driven applications such as knowledge graphs, ontology-guided information extraction, and GraphRAG-based caregiver-support systems. These applications have the potential to support informal caregivers and nursing professionals by providing structured, dementia-specific knowledge and guidance for managing agitation-related behaviours in real-world caregiving situations.

In the future, we plan to annotate additional textual data to further populate the EDEM-CONNECTONTO-based knowledge graph (eDEM-KG). We also intend to incorporate additional data sources, including semi-structured interviews and focus group discussions with informal caregivers and relatives of PwD. These sources will enable the refinement of relational and contextual constructs within the ontology and ensure that it captures real-world caregiving dynamics beyond literature-derived concepts. Furthermore, publicly available caregiver-generated content (e.g., moderated online forums and support communities) will be systematically analysed and mapped to ontology classes and relations to support ontology enrichment through data-driven concept discovery. While EDEM-CONNECTONTO currently focuses on agitation-related behaviours, future extensions will broaden its scope to include additional behavioural and psychological symptoms of dementia (BPSD). This extension will follow established ontology engineering methodologies, including competency question formulation, expert validation, and iterative refinement.

## Data Availability

The EDEM-CONNECTONTO ontology is publicly available at the NCBO BioPortal at https://bioportal.bioontology.org/ontologies/EDEM-CONNECTONTO.

## Footnotes

1
https://bioportal.bioontology.org/ontologies/EDEM-CONNECTONTO

2
https://bioportal.bioontology.org/ontologies/EDEM-CONNECTONTO

3
https://www.healingwell.com/

4
https://huggingface.co/sentence-transformers/all-MiniLM-L6-v2

5
https://doi.org/10.5281/zenodo.17966383
