# Supplementary material for: eDEM-CONNECT: agitation ontology for the intelligent support of informal caregivers of people with dementia
Source: Front Aging. 2026 May 1;7:1780260. doi: 10.3389/fragi.2026.1780260 (PMC13176241; doi:10.3389/fragi.2026.1780260)
Supplement: Supplementary file 1 [file DataSheet1.pdf]

frontiers

eDEM-CONNECT: Agitation ontology for the intelligent support of informal caregivers of people with dementia: Appendix

Sumaiya Suravee [1,*], Christiane Pinkert [2] Iris Hochgraeber [3] Margareta Halek [4] Bernhard Holle [2,4] and Kristina Yordanova [1]

[1] *Institute of Data Science, University of Greifswald, Felix-Hausdorff-Straße 18 17489 Greifswald, Germany*
[2] *German Centre for Neurodegenerative Diseases (DZNE), 58453 Witten, Germany*
[3] *CIS - Cellitinneninstitut für Qualitätssicherung, Seniorenhaus GmbH der Cellitinnen zur hl. Maria, 50931 Cologne, Germany*
[4] *School of Nursing Science, Faculty of Health, Witten Herdecke University, Witten, Germany*

Correspondence*:
Sumaiya Suravee
sumaiya.suravee@uni-greifswald.de

RESULTS FROM LITERATURE REVIEW

In EDEM-CONNECTONTO, concepts were derived from 18 research articles, covering the target domain related to PwD. Several studies contributed substantially to the conceptual structure of the ontology. From Yordanova et al. Yordanova et al. (2017), five concepts:'Technical affinity', 'Type_1_user', 'Type_2_user', 'Type_3_user', and 'Medical history' were extracted, related to user profiling, including technical affinity and multiple user type classifications, as well as medical history. From IdA-assessment Bartholomeyczik and Halek (2010), the topographical characteristics of agitation in PwD were extracted, as well as relevant background information about the individuals. Besides, in the insideDEM project Kernebeck et al. (2019), IdA was evaluated for use by caregivers within a tablet-based application. During several expert workshops, the items were reviewed for their suitability in home-care settings, resulting in 56 IdA items being selected for this context. Based on these inclusion criteria, we extracted both topographical data and background information about the PwD from the IdA instrument. 20 concepts of 'Independence in activities of daily living', 'Lifestyle before dementia' and 'Mood and emotion' were extracted from this study. 34 sub-concepts of the higher-most concept 'Topography' were also extracted from the study Bartholomeyczik and Halek (2010). The CMAI model Cohen-Mansfield (2012) is widely used and has been validated across almost all home-care settings Kupeli et al. (2018); Koss et al. (1997). Moreover, its conceptualisation of agitation is considered universal, making it suitable for application across diverse care environments. 38 sub-concepts of 'Physical_aggressive',

’Physical_nonaggressive’, ’Verbal_aggressive’, ’Verbal_nonaggressive’ are conceptualized from the following CMAI study: Cohen-Mansfield and Libin (2005); Cohen-Mansfield (2008, 1997); Baillon et al. (2004); Zhang et al. (2020). Consequently, 6 sub-concepts of ’Strong emotion’ were extracted from the study DEMENTIA (2007). The deductive analysis of the literature was based on approaches to managing agitation of PwD, in particular the DICE approach Kales et al. (2015) and the IdA-assessment Bartholomeyczik and Halek (2010). After analysing the DICE approach Kales et al. (2015), 26 sub-concepts of ’Factors related to the people with dementia’, 27 sub-concepts of ’Factors related to the people with dementia’, 6 sub-concepts of ’Environmental factors’, and 18 sub-concepts of ’Caregiver factors’ were extracted from this study. 12 concepts of ’Consequences’ were extracted from the study in Kales et al. (2015); Kong (2005). In Cohen-Mansfield & Parpura-Gill Cohen-Mansfield and Parpura-Gill (2007), the Treatment Routes for Exploring Agitation (TREA) approach is applied and consequently, ’Sleep disorder’ and ’Pain of the person with dementia’ were extracted. From Kolanowski et al. Kolanowski et al. (2011),’Temperature’, ’Light level’ and ’Noise level’ were extracted. ’Change in routine’ is extracted from the study Lawlor et al. Lawlor and Sunderland (1994). 2 concepts: ’Phone call frequency’, ’Visitor visit frequency’ were extracted from the study in Rhodes-Kropf et al. (2011). Related to dyadic relationships, an adapted approach combining deductive and inductive coding was used with the SoCA-Dem theory Köhler et al. (2021) serving as an analytical framework Thomas and Harden (2008). ’Family-carer’ concept was extracted from the study Ward-Griffin et al. (2007). ’Job/employment’ and ’Living_situation’ were concepts extracted from the study: Kohler et al. and Thomas et al. Köhler et al. (2021); Thomas and Harden (2008). ’Gender’ concept was extracted from the study Braun et al. (2009). The concepts extracted from the literature were used as a framework for analysis. Additional concepts were developed inductively from the data. In addition, 18 qualitative interviews with family caregivers of PwD from the UK MARQUE study Laybourne (2019) have been analyzed using secondary analysis. The MARQUE project consisted of six work streams, the results of which should contribute to a better understanding of agitation and dementia, and the workstreams explored how agitation affects the relationship between relatives and PwD and what helps in supporting the PwD. Subsequently, 32 concepts were added as the sub-concepts of "Interventions" based on the data, collected from the interviews. For the secondary analysis of these interviews, the same analytical framework was used in the evaluation of the five interviews with family caregivers. It is to be noted that the study participants were recruited without any differentiation between race, sex, migration or ethnic background. The interviews were conducted during the COVID-19 pandemic, which made the process of recruitment very difficult, especially for the vulnerable group of family caregivers.

THE RESULTING EDEM-CONNECTONTO

As no single “correct” way or methodology for developing ontologies exists, we debugged and evaluated EDEM-CONNECTONTO with the domain experts. This iterative design process continued through the entire ontology development cycle. One of the higher-most concepts, "Profile" defines the collection of general factors that characterise an individual, describing long term conditions of the person along with the typical behavioural patterns and abilities. The concept "Profile" is categorised by "Profile of caregiver" and "Profile of people with dementia". Then "Profile of people with dementia" is further classified on the living conditions, activities,

emotions, health conditions, and abilities of the PwD (see Figure 1). "Agitation", another higher-most concept, is classified into 5 sub-classes: "Verbal_aggressive", "Verbal_nonaggressive", "Physical_aggressive", "Physical_nonaggressive" and "Strong emotion". "Verbal_agreesive" is further classified into "Cursing", "Making_verbal_sexual_advances" and "Screaming" and "Verbal_nonaggressive" is classified into four sub-classes such as "Complaining", "Negativism", "Repetitive_sentences_or_questions" and "Strange_noises".

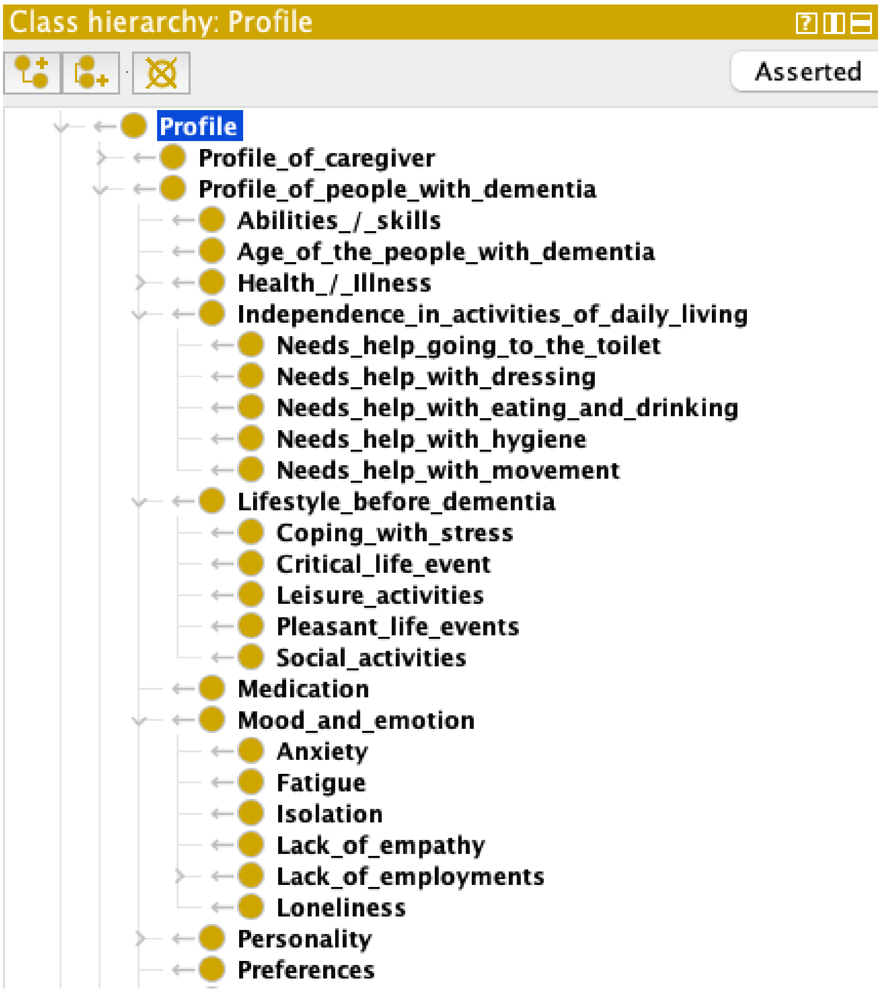

Figure 1. Subconcepts and sub-subconcepts of the upper-most concept “Profile” in the EDEM-CONNECTONTO

The factors that trigger agitation in PwD were presented in the higher-most concept "Causes". It has three subclasses, "Environmental_factors", "Interpersonal_factors" and "Factors_related_to_the_person_with_dementia". Each sub-class of "Causes" has 15, 28 and 30 sub-subclasses, respectively. Agitated behaviour of the PwD might lead to physical and psychological stress on the caregivers taking care of the PwD, such as heavy workload, disruptions to daily care routines, depression, and poor quality of life (see Cerejeira et al. (2012); Cubit et al. (2007); Feast et al. (2016)). Our domain experts identified these outcomes that the PwD might experience and added these outcomes as "Consequences" concept in the EDEM-CONNECTONTO. The

"Consequences" represent knowledge about the PwD might face challenges because of being agitated (for instance, agitated behaviour could lead to early placement in a nursing home). It has 12 direct subclasses. In general, family caregivers support the PwD to manage agitation to minimise the likelihood of adverse outcomes. Probable interventions are studied that could help the PwD to improve their conditions. The most hierarchical "Interventions" concept has 5 sub-classes, such as “Prevention”, “Problem solving”, “Acceptance”, “Reduction of negative consequences” and "De-escalation (see Figure 2). The last higher-most concept in EDEM-CONNECTONTO is "Topography", which describes the natural and artificial features of the surroundings when agitation occurs over a particular period of time. It has 5 direct sub-classes including "Attendees", "Duration", "Frequency", "Places" and "Situation" (see Figure 3). In total, these sub-classes have 29 sub-subclasses.

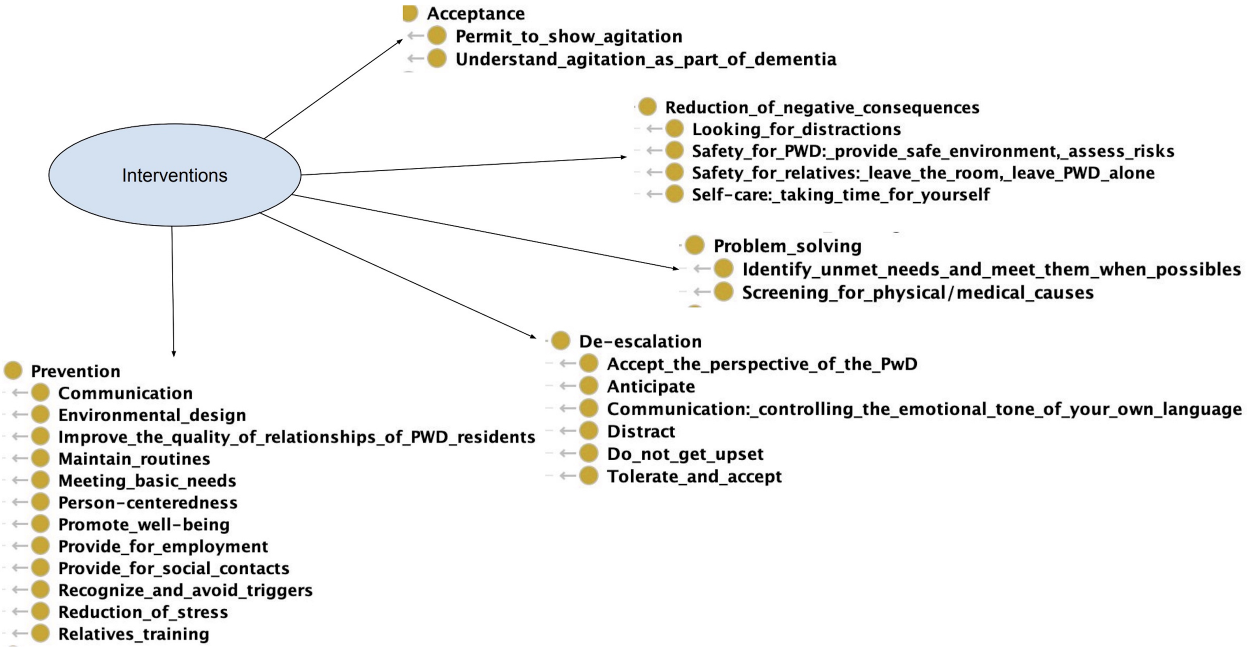

Figure 2. Identified concepts of the EDEM-CONNECTONTO concerning the possible interventions as their subconcepts.

REFERENCES

Baillon, S., Van Diepen, E., Prettyman, R., Redman, J., Rooke, N., and Campbell, R. (2004). A comparison of the effects of snoezelen and reminiscence therapy on the agitated behaviour of patients with dementia. *International journal of geriatric psychiatry* 19, 1047–1052

Bartholomeyczik, S. and Halek, M. (2010). *Assessmentinstrumente in der Pflege: Möglichkeiten und Grenzen* (Schlütersche)

Braun, M., Scholz, U., Bailey, B., Perren, S., Hornung, R., and Martin, M. (2009). Dementia caregiving in spousal relationships: A dyadic perspective. *Aging and mental health* 13, 426–436

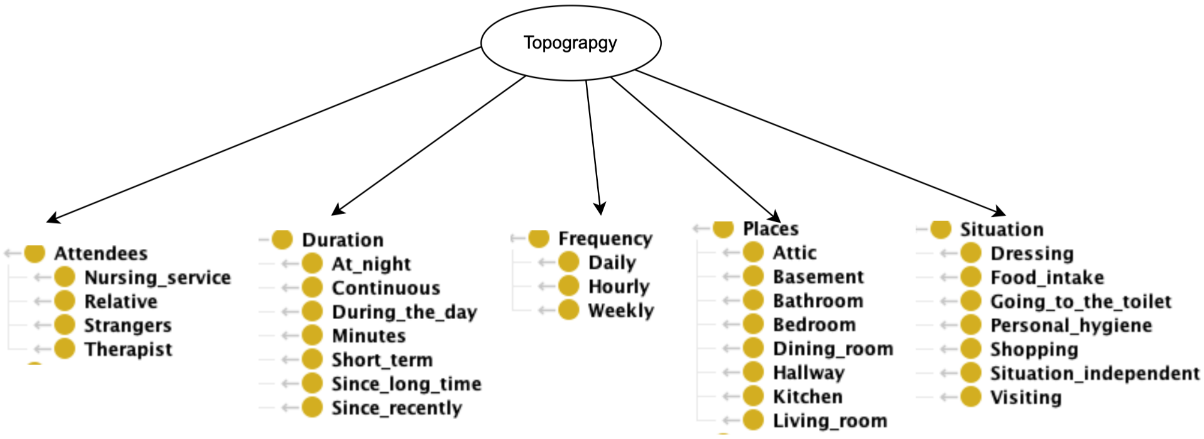

Figure 3. Subconcepts of the most hierarchical concept "Topography" in the EDEM-CONNECTONTO

Cerejeira, J., Lagarto, L., and Mukaetova-Ladinska, E. B. (2012). Behavioral and psychological symptoms of dementia. *Frontiers in neurology* 3, 73

Cohen-Mansfield, J. (1997). Conceptualization of agitation: results based on the cohen-mansfield agitation inventory and the agitation behavior mapping instrument. *International psychogeriatrics* 8, 309–315

Cohen-Mansfield, J. (2008). Agitated behavior in persons with dementia: the relationship between type of behavior, its frequency, and its disruptiveness. *Journal of psychiatric research* 43, 64–69

Cohen-Mansfield, J. (2012). Cohen-mansfield agitation inventory. *International Journal of Geriatric Psychiatry*

Cohen-Mansfield, J. and Libin, A. (2005). Verbal and physical non-aggressive agitated behaviors in elderly persons with dementia: robustness of syndromes. *Journal of Psychiatric Research* 39, 325–332

Cohen-Mansfield, J. and Parpura-Gill, A. (2007). Bathing: A framework for intervention focusing on psychosocial, architectural and human factors considerations. *Archives of gerontology and geriatrics* 45, 121–135

Cubit, K., Farrell, G., Robinson, A., and Myhill, M. (2007). A survey of the frequency and impact of behaviours of concern in dementia on residential aged care staff. *Australasian Journal on Ageing* 26, 64–70

DEMENTIA, W. I. (2007). Treatment of dementia and agitation: a guide for families and caregivers. *Journal of Psychiatric Practice* 13, 207

Feast, A., Orrell, M., Charlesworth, G., Melunsky, N., Poland, F., and Moniz-Cook, E. (2016). Behavioural and psychological symptoms in dementia and the challenges for family carers: systematic review. *The British Journal of Psychiatry* 208, 429–434

Kales, H. C., Gitlin, L. N., and Lyketsos, C. G. (2015). Assessment and management of behavioral and psychological symptoms of dementia. *Bmj* 350

Kernebeck, S., Holle, D., Pogscheba, P., Jordan, F., Mertl, F., Huldtgren, A., et al. (2019). A tablet app–and sensor-based assistive technology intervention for informal caregivers to manage the

challenging behavior of people with dementia (the insidedem study): protocol for a feasibility study. *JMIR research protocols* 8, e11630

Köhler, K., Dreyer, J., Hochgraeber, I., von Kutzleben, M., Pinkert, C., Roes, M., et al. (2021). Towards a middle-range theory of 'stability of home-based care arrangements for people living with dementia'(soca-dem): findings from a meta-study on mixed research. *BMJ open* 11, e042515

Kolanowski, A., Litaker, M., Buettner, L., Moeller, J., and Costa, P. T., Jr (2011). A randomized clinical trial of theory-based activities for the behavioral symptoms of dementia in nursing home residents. *Journal of the American Geriatrics Society* 59, 1032–1041

Kong, E.-H. (2005). Agitation in dementia: concept clarification. *Journal of advanced nursing* 52, 526–536

Koss, E., Weiner, M., Ernesto, C., Cohen-Mansfield, J., Ferris, S. H., Grundman, M., et al. (1997). Assessing patterns of agitation in alzheimer's disease patients with the cohen-mansfield agitation inventory. *Alzheimer Disease & Associated Disorders* 11, 45–50

Kupeli, N., Vickerstaff, V., White, N., Lord, K., Scott, S., Jones, L., et al. (2018). Psychometric evaluation of the cohen-mansfield agitation inventory in an acute general hospital setting. *International journal of geriatric psychiatry* 33, e158–e165

Lawlor, B. A. and Sunderland, T. (1994). A strategy for coping with behavioral changes. *Consultant* 34, 43–47

Laybourne, A. (2019). Managing agitation and raising quality of life: Semi-structured interviews with family carers of people living with dementia. Mendeley Data, Version 1

Rhodes-Kropf, J., Cheng, H., Castillo, E. H., and Fulton, A. T. (2011). Managing the patient with dementia in long-term care. *Clinics in geriatric medicine* 27, 135–152

Thomas, J. and Harden, A. (2008). Methods for the thematic synthesis of qualitative research in systematic reviews. *BMC medical research methodology* 8, 45

Ward-Griffin, C., Oudshoorn, A., Clark, K., and Bol, N. (2007). Mother-adult daughter relationships within dementia care: A critical analysis. *Journal of Family Nursing* 13, 13–32

Yordanova, K., Koldrack, P., Heine, C., Henkel, R., Martin, M., Teipel, S., et al. (2017). Situation model for situation-aware assistance of dementia patients in outdoor mobility. *Journal of Alzheimer's Disease* 60, 1461–1476

Zhang, Z., Yu, P., Chang, H. C., Lau, S. K., Tao, C., Wang, N., et al. (2020). Developing an ontology for representing the domain knowledge specific to non-pharmacological treatment for agitation in dementia. *Alzheimer's & Dementia: Translational Research & Clinical Interventions* 6, e12061
